# User Acceptance of a Home Robotic Assistant for Individuals With Physical Disabilities: Explorative Qualitative Study

**DOI:** 10.2196/63641

**Published:** 2025-01-13

**Authors:** Linda Sørensen, Dag Tomas Sagen Johannesen, Helinä Melkas, Hege Mari Johnsen

**Affiliations:** 1 Department of Health and Nursing Science Faculty of Health and Sport Sciences University of Agder Kristiansand Norway; 2 Lappeenranta-Lahti University of Technology Industrial Engineering and Management LUT School of Engineering Science Lahti Finland

**Keywords:** physical artificial intelligence, physical AI, health care robotics, assistive technology, content analysis, qualitative, health care, robotics, assistive, robot interaction, physical disabilities, readiness, amputations

## Abstract

**Background:**

Health care is shifting toward 5 proactive approaches: personalized, participatory, preventive, predictive, and precision-focused services (P5 medicine). This patient-centered care leverages technologies such as artificial intelligence (AI)–powered robots, which can personalize and enhance services for users with disabilities. These advancements are crucial given the World Health Organization’s projection of a global shortage of up to 10 million health care workers by 2030.

**Objective:**

This study aimed to investigate the acceptance of a humanoid assistive robot among users with physical disabilities during (1) AI-powered (using a Wizard of Oz methodology) robotic performance of predefined personalized assistance tasks and (2) operator-controlled robotic performance (simulated distant service).

**Methods:**

An explorative qualitative design was used, involving user testing in a simulated home environment and individual interviews. Directed content analysis was based on the Almere model and the model of domestic social robot acceptance.

**Results:**

Nine participants with physical disabilities aged 27 to 78 years engaged in robot interactions. They shared their perceptions across 7 acceptance concepts: hedonic attitudes, utilitarian attitudes, personal norms, social norms, control beliefs, facilitating conditions, and intention to use. Participants valued the robot’s usefulness for practical services but not for personal care. They preferred automation but accepted remote control of the robot for some tasks. Privacy concerns were mixed.

**Conclusions:**

This study highlights the complex interplay of functional expectations, technological readiness, and personal and societal norms affecting the acceptance of physically assistive robots. Participants were generally positive about robotic assistance as it increases independence and lessens the need for human caregivers, although they acknowledged some current shortcomings. They were open to trying more home testing if future robots could perform most tasks autonomously. AI-powered robots offer new possibilities for creating more adaptable and personalized assistive technologies, potentially enhancing their effectiveness and viability for individuals with disabilities.

## Introduction

### Background

Health care is evolving to adopt a more proactive approach through the principles of personalized, participatory, preventive, predictive, and precision-focused services (P5 medicine). This approach to patient-centered care uses technologies such as robots. Artificial intelligence (AI) is central in the development of these robots to enhance care and treatment processes [1]. AI refers to “the application of computer technology to mimic human intelligence and critical thinking” [2]. Through machine learning, AI systems can autonomously enhance their algorithms by learning from data and cognitive inputs without specific programming. This research field is rapidly expanding and becoming increasingly interdisciplinary, attracting a growing number of researchers.

AI techniques can enhance the management of comprehensive health services by supporting medical professionals such as physicians, nurses, and administrators [3]. For example, AI can provide timely access to medical information, streamline hospital operations, and ensure patient safety by improving medication management and care engagement [4]. Furthermore, AI may optimize logistics, such as the just-in-time delivery of drugs and equipment, and improve training for health care personnel, potentially reducing disparities between urban and rural health care services [3]. Furthermore, AI systems have shown promising results within health care in predicting conditions such as sepsis, cancer, and cardiovascular risk and monitoring vital signs in intensive care units [5-7].

Physical AI (PAI) [8-10] encompasses the application of AI technologies within physical systems, devices, and environments, enabling the performance of tasks that involve physical interaction or manipulation in the real world. In contrast to software-based AI, which functions in digital settings, such as data analysis, natural language processing, and web-based recommendation systems, PAI is evident in concrete, real-world applications. Examples of PAI include robotics, intelligent sensors, autonomous vehicles, and other cyberphysical systems that merge computational intelligence with physical functionalities [8-10].

AI-powered robotics in health care may offer a range of potential benefits that could revolutionize the field [11]. These systems provide precision and consistency in tasks, often surpassing human capabilities. Precision is particularly vital in surgeries to avoid adverse events and harm to patients. AI-powered robotics also facilitate telemedicine, allowing health care professionals to conduct consultations and services remotely, thus extending medical services to underresourced areas [12]. Such developments are crucial as the World Health Organization has highlighted global staffing challenges, projecting a potential shortage of up to 10 million health care workers by 2030 [13]. To address these challenges, the health care sector is increasingly adopting robotics and AI to mitigate workforce shortages. AI-powered robots may not only support complex medical procedures but could also assist in daily care activities, potentially reducing the workload on health care staff. In addition, robots integrated with AI could play a pivotal role in personalizing, adapting, and improving services to users with disabilities [12]. Despite the high initial costs of implementing AI-powered robotics in health care, the long-term benefits, streamlined processes, and overall cost savings could significantly improve the efficiency and accessibility of health care delivery [14].

### Related Work

The field of scientific robotics is advancing the development of social assistive robots capable of adapting to local social norms, interpreting human emotions and desires, and learning by emulating human behavior. While these socially assistive robots primarily offer companionship and facilitate basic exercises, their capabilities in carrying out a variety of physical assistance tasks, such as carrying and delivery, are still limited. Robots able to assist people physically (carrying and delivery) include robots such as Care-O-bot, Doro, TIAGo, Lio, EVE, and Hobbit. They can be remotely controlled via home services or preprogrammed to operate autonomously, performing tasks primarily ordered through a tablet [15-17].

Research indicates that older adults generally perceive socially assistive robots as enjoyable, friendly, and safe [18,19]. However, studies by Wu et al [20] and Lee et al [21] highlight that older individuals without functional impairments find little relevance in these robots. The domain of assistive robotics for individuals with physical disabilities, whether young or old, remains nascent. Sørensen et al [22] concluded in their review that robots designed to assist with a variety of physical tasks currently demonstrate an inadequate level of technical readiness for home use, particularly due to their lack of personalization and overall preparedness for everyday assistance. A key challenge for effective assistive robots is managing a wide variety of assistive situations and tailoring robots’ interactions to the living contexts and preferences (or habits) of the individuals they assist [23]. So far, assistive robots that provide physical assistance have had limited capabilities for detailed personalization, which is essential for effectively serving their users [15]. However, recent advances in AI may enable these robots to adapt to their users’ environments and offer services in a more personalized manner [22].

Previous studies highlight that robot personalization is crucial for user acceptance as it aligns robots with the users’ preferences and needs, thereby enhancing their willingness to use them [19,24,25]. Rather than taking over enjoyable activities that contribute to physical activity, robots should assist with challenging or energy-draining tasks, thus allowing users to focus on preferred activities [15]. However, personalization can be costly and time-consuming and requires ongoing technical support as users’ needs evolve with their health status. Given these challenges, multidisciplinary research, including participatory design in PAI, is essential [26]. PAI combines insights from technical and human sciences to develop robots that intelligently interact with their physical environment, adapt to malfunctions, and integrate with existing systems. This approach fosters innovation and ensures that robots are practical, safe, and effective in dynamic real-world settings [4].

Currently, there is a lack of knowledge about user acceptance of robots able to assist physically or of PAI among individuals with physical disabilities. In this paper, we present the results of a multidisciplinary study investigating individuals with physical disabilities’ acceptance of a humanoid robot (EVE) providing personalized assistance in a simulated home setting.

### Theoretical Frameworks for Assessing Acceptance of Technology

In total, 2 models have frequently been used to assess acceptability in users of assistive and social robots: the Almere model [27] and the model of domestic social robot acceptance (DSRA) [28]. The Almere model by Heerink et al [27] extends the unified theory of acceptance and use of technology [29] by including factors related to social interaction. The model’s effectiveness has been evaluated through controlled experiments and longitudinal data collection involving 3 different assistive social robots in older adult care facilities and homes. The findings demonstrated significant support for the model and, thereby, contribute to the understanding of the factors influencing the acceptance of assistive social robots among older users. Heerink et al [27] highlight the importance of considering both functional and social variables in the design and implementation of these technologies.

DSRA [28] is rooted in the theory of planned behavior and extends it by incorporating factors specific to the context of domestic social robotics. These include utilitarian and hedonic attitudes, social and personal normative beliefs, and control beliefs. The study’s [28] findings underscore the importance of considering these factors in the design and development of social robots to ensure their acceptance and integration into domestic settings.

In this study, we used these models as frameworks in a qualitative investigation of acceptance in potential future users of humanoid robots that are able to assist their users physically. We use the term *physically assistive robots* in this paper.

### Objectives

People with physical disabilities, whether from disease or injury, encounter obstacles in their everyday activities and social participation. Many rely on formal or informal caregivers for assistance with activities of daily living. Upcoming demographic shifts pose significant challenges to the availability of home care services and assistants, potentially affecting individuals’ ability to access necessary support [15].

The aim of this study was to investigate the acceptance of the prototype humanoid assistive robot EVE in individuals with physical disabilities during (1) AI-powered (using a Wizard of Oz methodology [30,31]) robotic performance of predefined personalized assistance tasks and (2) operator-controlled robotic performance.

In addition, we explored potential users’ perspectives on how physical robotic assistance might impact their independence and autonomy.

On the basis of the models used as frameworks, we also suggested changes to the concepts we found more prominent when investigating acceptance of robots primarily designed for physical assistance rather than social interaction.

## Methods

### Study Design

This study used an explorative qualitative design, with data collection consisting of user testing in a simulated home environment and individual interviews. We used human-centered design (HCD), a creative approach to problem-solving that starts with understanding the needs of the people one is designing for and ends with solutions tailored to their needs. HCD emphasizes incorporating the end user at the center of the design process, ensuring that products or services are tailored to meet their needs and enhance usability [15,32]. The process is iterative, involving multiple stages of prototypes and tests to refine and validate the service based on user feedback, ensuring that the service is usable in real-world scenarios [33]. As recommended for HCD, we worked iteratively and in a multidisciplinary manner with robot engineers; robot operators; and, most importantly, the potential future users we targeted—individuals with physical disabilities [34]. We define usability as “the extent to which a system, product or service can be used by specified users to achieve specified goals with effectiveness, efficiency and satisfaction in a specified context of use” (ISO9241) [35].

Before the user test, we asked the participants for which tasks they would find it most useful to receive physical assistance from a robot. We then shared these insights with the robot engineers, enabling the robot operators to practice performing these specific tasks and make necessary adjustments to align with the robot’s current technical capabilities.

First, user testing [36] of the robot was conducted in a simulated home environment, allowing participants to interact with the robot in a realistic setting. This was followed by video-stimulated interviews with the participants approximately 1 week later, providing them with an opportunity to reflect on their experiences. For data collection and analysis, we used a directed approach [37] based on the 2 theoretical frameworks: the Almere model and the model of DSRA. These frameworks guided the evaluation of how users with disabilities accepted robot home assistance.

### The Robot

**Figure 1:**
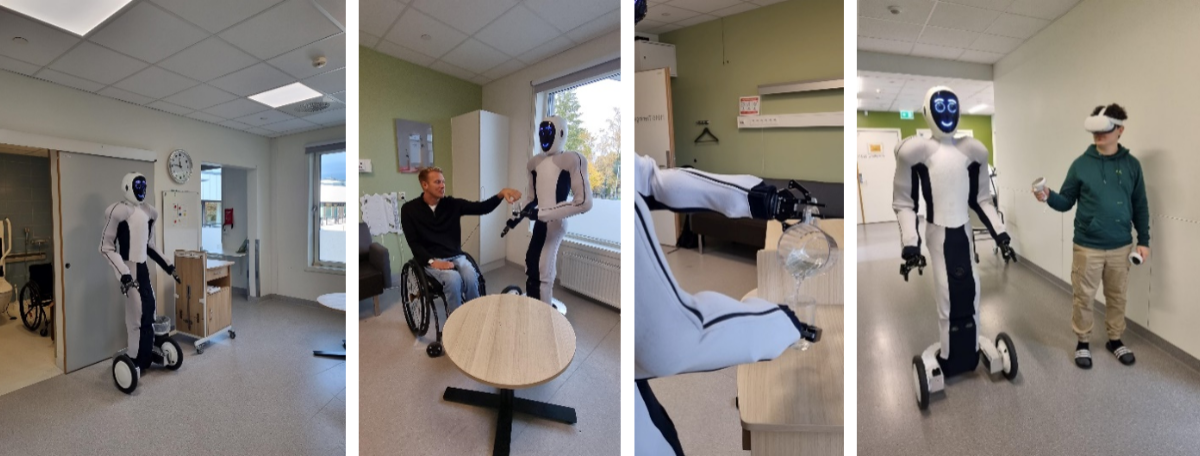
The humanoid robot EVE in the user testing scenario (pictures by LS).

This project is part of a broader project conducted at the University of Agder focusing on exploring humanoid robotic applications in health care. The university has acquired a state-of-the-art humanoid robot named EVE (Figure 1) specifically for research purposes.

EVE is a full-size humanoid robot designed for research and logistic applications. Standing at a height of 186 cm and weighing 80 kg, EVE maneuvers on 3 wheels, equipped with grippers enabling it to manipulate objects of varying sizes. The robot is equipped with cameras and sensors to perceive and interact with its surroundings. Its mobility, dexterity, and balance allow it to navigate complex environments and manipulate objects effectively [38].

The robots’ AI-powered movements are trained using a diverse dataset from 30 EVE robots, enabling it to perform a wide range of tasks, from tidying to social interactions. This base model is then fine-tuned for specific capabilities, such as door manipulation or warehouse tasks. Using data instead of code, robot operators train (using virtual reality glasses and controllers) and expand the robot’s abilities to increase flexibility in the robot’s functions [38].

**Figure 2:**
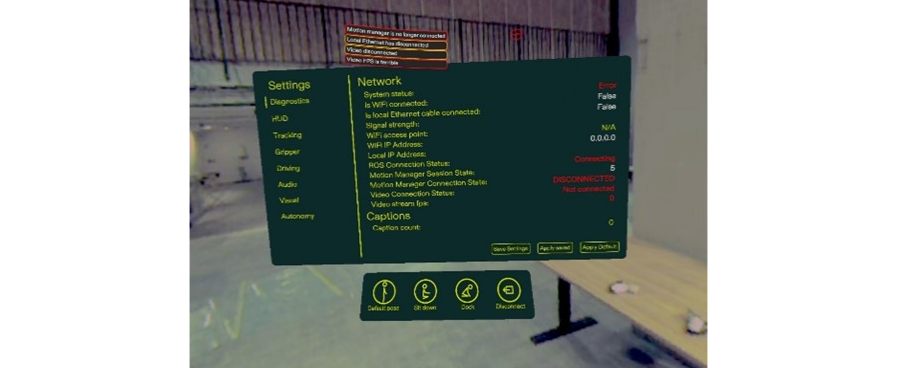
Sync/Robot Control (picture by 1X Technologies, reused with permission).

In this study, the robot was controlled using Meta Quest 3 glasses. Operators were given a stereoscopic view through 2 fish-eye lenses calibrated to provide depth perception. The Meta Quest 3 controllers were used to manipulate the robot’s wrist, whereas an algorithm (self-developed Sync/Robot Control; Figure 2) translated these movements into more complex actions, enabling the robot to mimic human motion based on wrist inputs.

### Recruitment

Participants were recruited through purposive sampling by coordinators of health services at a spinal cord ward at a rehabilitation hospital in the eastern region of Norway and a municipality in the southern region of Norway. The inclusion criteria for this study were individuals aged ≥16 years with physical disabilities, living at home, and receiving formal or informal care.

The coordinators suggested relevant candidates to the researcher based on the inclusion and exclusion criteria (Textbox 1). The first author, LS, then contacted the candidates with information about the study with an invitation to participate. Seven of the participants had previously taken part in a focus group activity [15] where they received information about the robot and saw videos of the robot performing different activities before their interaction with it (although not in the context of health care). The 2 remaining participants received information about the robot and watched the same videos after agreeing to take part in this study.

**Inclusion and exclusion criteria.**

**Inclusion criteria**

- Aged ≥16 years
- Having physical disabilities requiring physical assistance with personal care or activities of daily living

**Exclusion criteria**

- Cognitive challenges or language challenges that would make commanding the robot or answering questions in Norwegian difficult

### Data Collection and Procedure

The user tests took place in a hospital room designed as an apartment that is used for patients to practice becoming independent before discharge. The apartment has a living room with a table, chair, closet, and television. It also includes an accessible kitchen and bathroom. The control room for controlling the robot through virtual reality was set up in the adjacent room. It included live video feeds displayed on a screen, live audio from the robot, and an additional speaker system (Jabra speakers) to enhance sound quality. The participant-robot interaction was video recorded using 2 cameras in the room (Multimedia Appendix 1). The participants’ interaction with the robot had 3 parts (Textbox 2). First, participants watched EVE perform 5 tasks. The tasks were identified as useful in a previous study investigating users with physical disabilities’ needs for assistance [15]. We used a Wizard of Oz approach [30,31], in which EVE appeared to perform tasks autonomously while a human operator discreetly controlled the robot behind the scenes unbeknownst to the participants to simulate its functionality. This technique is often used in developing and testing technologies such as health care robots and enables researchers to evaluate interactions, collect user feedback, and identify improvement areas before fully automating the system [30,31,39]. The presence of the operator was revealed to participants at the conclusion of the study.

Second, the participants were instructed to ask the robot to bring a drink of water. Before the user testing, LS had asked each participant how they would need and prefer to have a drink of water served. After the participants had asked the robot to bring the water, the robot nodded for confirmation and went to the kitchen to bring water (Wizard of Oz methodology). Details of the personalized service are described in Textbox 2.

In the third part of the interaction, the participants were instructed to call the operator of the robot to request assistance with a chosen task. This part was designed to simulate a scenario in which a remote operator could control the robot for tasks that were not yet automated (human in the loop). Participants selected tasks that they often needed help with, such as picking up an item from the floor (Textbox 2). The participant first called out “Hallo,” prompting the operator to connect to the robot via voice and ask how they could assist. After retrieving the requested item, the operator asked (through the robot) if any further assistance was needed before saying, “just call again if there is anything you need. Have a nice day.” The robot then returned to its parking space, which was chosen by the participant. Some preferred the robot to be parked in the living room, whereas others wanted it out of sight, in which case it returned to the kitchen. The user test was facilitated by LS and the second author, DTSJ, in collaboration with the operators of EVE.

Approximately 1 week after the interaction, participants were interviewed at a time and place of their choosing. Seven interviews took place in the participants’ homes, whereas 2 were conducted at a hospital due to the participants’ short-term admissions. The first author used video-stimulated recall, a retrospective think-aloud interview technique, to help participants recall, discuss, and reflect on their robot interaction [40].

The interviews were conducted by LS, an occupational therapy specialist with knowledge of the participants’ functional impairments and daily challenges but no clinical relationship with them. The semistructured interviews were guided by acceptance concepts from the Almere model and the model of DSRA, with additional questions focusing on independence and autonomy (Multimedia Appendix 2).

**The robot-participant interaction in 3 parts.**

**Demonstration of robot tasks**

- Tidying up, including picking up trash from the table and placing it in the garbage bin
- Retrieving a towel from the floor and placing it in the laundry basket in the bathroom
- Pulling down a roller curtain using a string
- Verbally announcing the day’s schedule
- Opening a bag of potato chips and pouring them into a bowl

**Bringing the participant a drink of water, tailored to each user’s specific needs**

Four participants received a water bottle that the robot obtained from the fridge:

- For one participant, the robot placed the water bottle on the table (participant 6).
- Three participants received the water bottle in their hand (participants 1, 2, and 7). One participant had paralysis, which required the robot to hold the bottle still for some time until the participant managed to get a grip around the bottle. The robot released the grip as the participant said thank you.
- One participant who also had paralysis had a glass delivered to his hand in a similar way (participant 3).
- Two participants had their glasses placed on a table in front of them. The robot then filled a mug of water in the kitchen, came to the living room, and filled the glass of water. One was a regular glass (participant 4), and the other was a wineglass. This participant preferred a wineglass to be able to grip and hold it by the stem (participant 8).
- One participant who was not able to move their arms had the robot deliver a glass with a straw to a stand in front of their face that was mounted on the electric wheelchair (participant 5).
- One patient was served the drink of water in a bottle with a straw and drank from the straw as the robot held the bottle (participant 9).

**A task of the participants’ own choosing**

- Picking up an object that was dropped on the floor (participants 1, 8, and 9)
- Bringing the participant a newspaper (participant 6)
- Bringing a blanket to lay over the participant’s legs (participant 2)
- Taking something out and down from a top cupboard in the kitchen (participant 7)
- Taking out a bag of snacks from a kitchen drawer, opening it, and bringing it to the participant (participant 3)
- Scratching the back of the participant’s head using a scratching stick (participant 5)
- Bringing the television remote control and placing it in the participant’s lap (participant 4)

### Analysis

The interviews were transcribed verbatim by the first author, omitting minor speech hesitations to improve readability. For the analysis, we used the 16-step process described by Assarroudi et al [41] in a combination of deductive and inductive approach [37,42]. First, in the directed approach, the data were coded deductively using a structured categorization matrix [41] based on the predetermined codes from the Almere model and the model of DSRA (Multimedia Appendix 3 [43-51]). According to Hsieh and Shannon [37], directed content analysis is useful when a theory exists about a phenomenon that needs further refinement or development through qualitative research [37]. Second, inductive coding was performed to search for additional concepts that might affect the acceptance of the assistive robot and concepts that needed to be modified or could be excluded. All data analysis was performed using the NVivo software application (version 14; Lumivero). The initial coding was performed by LS. An extract from the coding was then reviewed by and discussed with all authors until an agreement was reached about categories and subcategories. The findings were finally organized into 7 categories with 13 subcategories. Table 1 shows an example from the analysis process.

**Table 1:** Example of the analysis process.

Participant statement	Codes	Category
“I tend to drop my phone on the floor. If the robot could pick it up, that would definitely be a useful task for me.”	Pick up an item	Usefulness
“...no, giving it verbal commands would not be hard for me, if you have a pet you’re quite used to it.”	Voice commanding; not perceived difficult	Ease of use
“I wonder a bit about the monitoring, if people can see and hear what’s happening in my house through the robot, I wouldn’t like that.”	Distant controlling Monitoring	Privacy

### Ethical Considerations

All methods were carried out in accordance with relevant guidelines (Helsinki Declaration) and national laws. This study was approved by the Research Ethics Committee at the Regional Committees for Medical and Health Research Ethics (467937), the University of Agder- Norway's ethical board, and Sikt—Norwegian Agency for Shared Services in Education and Research (343362). All participants gave their written informed consent to take part in the study, with the possibility to withdraw at any time. To ensure confidentiality and anonymity throughout the analysis, identifiable details in the interviews were anonymized, and participants and other mentioned persons were assigned numbers. As the robot continuously records picture and sound input and stores these data, participants signed an additional consent form for this data collection. All data stored in the robot were deleted 1 week after the robot-participant interaction. Participants were not compensated for participation, but travel expenses were compensated.

## Results

### Overview

Eleven persons (Table 2) with physical disabilities (3 women, 8 men) agreed to participate in the user tests. Of these 11 individuals, 2 withdrew (1 woman and 1 man), 1 because of illness and 1 because the assistant called in sick that day. The participants were aged between 27 and 78 years, with a median age of 59 years. One participant had muscular dystrophy, 7 had a cervical spinal cord injury (SCI; paralysis to arms and legs), 1 had a thoracic SCI (paralysis to the legs), and all were wheelchair users (full time).

**Table 2:** Participant characteristics.

Participant ID	Sex	Age (years)	Diagnosis	SCI^a^ level	Assistance (h/wk)	Source of assistance
1	Male	69	SCI	C5-6	31.5	Assistants and partner, parents, or children
2	Male	42	SCI	C5-6	3	Home nursing services; assistants; partner, parents, or children; and friends
3	Male	37	SCI	C6-7	45	Home nursing services; assistants; partner, parents, or children; and friends
4	Male	27	SCI	C5	91	Home nursing services; assistants; partner, parents, or children; and friends
5	Male	59	SCI	C3	154	Assistants; partner, parents, or children; and friends
6	Male	78	SCI	Th4-5	31.5	Home nursing services and partner, parents, or children
7	Male	31	SCI	C5	52	Home nursing services; assistants; partner, parents, or children; and friends
8	Female	78	SCI	C6	90.5	Home nursing services; assistants; and partner, parents, or children
9	Female	60	Muscular dystrophy	Lim girdle	168	Assistants; partner, parents, or children; and friends

^a^SCI: spinal cord injury.

Although many of the participants had the same diagnosis, the group was heterogenous in terms of level of independence. Table 3 shows the participants’ level of independence according to the Barthel Index for Activities of Daily Living [52], which assesses independence on a summarized scale from 0 to 20, where 20 is independent. All participants received various degrees of assistance with activities of daily living (Table 3). All participants considered themselves above average in being familiar with technology, such as smart house technology, computers, apps, and technical assistance devices. Two participants lived in accessible apartments (one rented and one owned). Seven of the participants lived in private accessible houses that were adapted for their particular needs, with automatic doors or windows, wider doors, ramps, elevators, and no thresholds.

**Table 3:** Barthel index^a^.

Participant ID	Feeding (0-2)	Bathing (0-1)	Grooming (0-1)	Dressing (0-2)	Bowels (0-2)	Bladder (0-2)	Toilet use (0-2)	Transfers (0-3)	Mobility (0-3)	Stairs (0-2)	Total (0-20)
1	1	0	1	1	2	2	2	2	2	0	13
2	1	0	1	1	2	2	0	2	1	0	10
3	1	0	1	1	2	2	0	2	1	0	10
4	0	0	0	0	0	0	0	0	0	0	0
5	0	0	0	0	0	0	0	0	1	0	1
6	2	0	0	0	0	0	0	3	1	0	6
7	2	0	1	2	2	2	0	3	1	0	13
8	2	0	1	0	0	0	0	0	0	0	3
9	0	0	0	0	0	0	0	0	0	0	0

^a^The Barthel Index for Activities of Daily Living assesses the level of independence on a scale from 0 to 20, where 20 is independent.

The extracted main categories based on the concepts from the Almere model and the model of DSRA were hedonic attitudes, utilitarian attitudes, personal norms, social norms, control beliefs, facilitating conditions, and intention to use (definitions can be found in Multimedia Appendix 3). These concepts with subcategories are shown in Table 4. The results related to each category and subcategory will be presented in the following sections. We made some smaller changes to the subcategories from the theory based on the statements from the participants. This is further elaborated on in the Discussion section.

**Table 4:** Acceptance model categories and subcategories—used, modified, excluded, and added.

Category	Hedonic attitudes	Utilitarian attitudes	Personal norms	Social norms	Control beliefs	Facilitating conditions	Intention to use
Subcategories used	Enjoyment	Perceived usefulness Ease of use Perceived adaptiveness	Privacy Trust	Social influence Perceived sociability Societal impact	Anxiety Safety	No subcategories	No subcategories
Modified subcategories used	Attractiveness Appearance, including animacy	—^a^	—	—	—	—	—
Excluded subcategories	Social presence Companionship	—	—	Status	Self-efficacy Cost	—	—
Added subcategories	—	—	Independence Autonomy	—	—	—	—

^a^Not applicable.

Altogether, 573 statements were coded from the transcripts. Of these 573 statements, 109 (19%) were used more than once as they fit in more than one subcategory. Of the subcategories, the participants spent most time talking about the robot’s functions and how it could assist with their needs. The usefulness category included 123 statements, with the most frequently mentioned tasks being picking up items dropped on the floor and bringing a drink of water. We used the International Classification of Functioning, Disability, and Health (ICF) to organize the findings (Table 5). The ICF is a framework developed by the World Health Organization for measuring health and disability at both the individual and population levels. Other authors have previously used the ICF concerning socially assistive robotics to evaluate assistive technologies’ impact on users’ lives and group activities that humans perform [15,53,54].

**Table 5:** Assistive tasks that would make the robot useful.

ICF^a^	Useful assistance categories and tasks
Activity and participation	Bringing items Water Snacks Preheated food Medication Blanket Newspaper Glasses Urine drainage bag Phone Clothes Shoes Remote control Picking up an object dropped to the floor Calendar reminders Assisting with transfers Emergency alerts Housework Tidying Wash windows Putting things in or taking things out of the dishwasher Assisting with technical aids Moving technical aids; bringing, for example, wheelchair, toilet chair, or walking frame Putting on tarpaulin Changing the position of the bed Controlling the lift and car lift Opening doors Charging phones or tablets
Self care	Scratching Putting on or taking off a comforter

^a^ICF: International Classification of Functioning, Disability, and Health.

### Hedonic Attitudes

#### Appearance (Attractiveness) and Animacy

The participants’ statements about the robot did not relate to its attractiveness and, thus, did not fit the predefined subcategory of attractiveness from the model of DSRA. However, they were concerned with how the robot should look and its human likeness. Therefore, we named this subcategory Appearance and Animacy. Half of the participants perceived the robot as overly large and described it as bulky and masculine. Others valued the robot’s ability to reach levels inaccessible to them, stating that this was particularly beneficial for those using wheelchairs. Participants offered no specific comments about the robot’s hands, which featured very mechanical grippers. They did not express a preference against more humanlike fingers, recognizing the functionality of the grippers as paramount. All but 1 participant appreciated the robot’s humanlike appearance. They did not desire a greater human likeness, explaining that they preferred it to retain its robotic nature to avoid a “creepy” overly humanlike appearance:

They have made a friendly design on it and stylish in relation to color and so you should not make it more human-like than that really. There is something about the fact that with a robot being a robot...you should not pursuit more resemblance to humans, but in relation to meeting a robot it’s easier if it looks like a human than not at all. But maybe not too much.

Participant 2

However, they were interested in the robot having a humanlike voice and the option to personalize this voice according to their preferences. While some participants appreciated the robot’s mobility on wheels, others expressed a preference for a smaller, lighter robot with legs capable of walking in stairs. One participant expressed a wish for the robot to have a humanlike mask and even hair:

No, first of all, it could have had completely different colors, been a little neater, smaller, not so dominant in the surroundings and then I think maybe you could also make a more human mask, get hair on the head, more human details, as human as possible for a robot.

Participant 1

#### Enjoyment

As the primary focus of the user interaction was the robot providing practical assistance and not being a social figure, we did not particularly ask about the participants’ level of enjoyment. Still, 3 participants mentioned that they enjoyed the encounter and that they believed that it would be fun to have a robot assisting them:

I’ve actually always liked [technology] and I liked it better 10-20 years ago than I do now...I think this is great fun, but it will probably also vary, but I just think it would be fun.

Participant 9

Another participant stated the following:

...yes, it’s very funny this robot helping out, this is the future, but it still seems far ahead...I thought it had come further. It would have been really fun to test the robot when it was fully AI powered. I’m very excited to see how smart it will be to understand its surroundings.

Participant 2

### Utilitarian Attitudes

#### Perceived Usefulness

All except 1 participant expressed being impressed by the robot’s functionality, particularly as the robot picked up a bag of chips, opened the bag, poured it into a bowl without spilling anything, and then served the bowl to the participants. During this task, the participants could see both finer and grosser movement skills from the robot. The one participant being most skeptical referred to the robot as an early-stage prototype, relating this mainly to its speed:

No, putting aside that I found it looking scary, I thought it might be only the second version at best, and its movements were very slow.

Participant 1

Another participant stated the following:

I was skeptical about what he could pick up...but I was very impressed when he picked up that bag, and no spilling at all, then I was very impressed with it, yes thought it worked really well.

Participant 6

All the participants mentioned that tasks such as tidying, bringing them things, and picking up things they dropped on the floor would be particularly beneficial for them. The tasks that they chose for the interaction with the robot were of such character and were personalized to each participant’s needs (Textbox 2). The participants appreciated that the robot could serve a drink specifically aligned to their needs and respond to their commands for a particular action. Several of the participants chose for the robot to pick up an item from the floor. Most of the participants were not able to do this if they, for example, accidentally dropped their phone or other items.

The participants mentioned other assistance tasks that would make the robot useful for them: turning lights off and on, answering the door, reading and answering mail, helping write signatures and other notes, scratching when itching, attaching iPads to charging cords, putting cutlery in and out of the dishwasher, serving guests with drinks and snacks, turning on and filling the washing machine, adjusting the comforter if they were too hot or cold, supporting them when transferring to the toilet, and adjusting the electric bed.

The interaction with the robot also led the participants to expect more advanced tasks, including tasks that would require the robot to function outside the home. A few participants mentioned assistance with their technical aids. One expressed that, if the robot could follow him to his car and then roll his walking frame back to the apartment, that would be very useful for him. Taking the tarpaulin off his outdoor electric wheelchair and then putting it back on when he returned would also be highly beneficial. One participant wished for the robot to be her walking partner. She wanted the robot to assist with minor repositioning of her hand so that she could control the joystick of her wheelchair without fear of her hand falling off the armrest. Another participant wanted the robot to control his car lift for more effective boarding, and one suggested that the robot could serve him water outside on his veranda during the summer.

Apart from physical assistance tasks, all but 1 participant appreciated that the robot reminded them of their agenda. They suggested integrating the software with their phones, noting that registering appointments and activities in multiple digital calendars would be cumbersome:

That [robot giving reminders] would have been useful for me, just ask my wife (laughter).

Participant 4

During the user test, the participants interacted with the robot both in an automated mode (using a Wizard of Oz methodology) and a mode in which the robot was operator controlled, simulated as “municipality services-personnel” controlling the robot. They found the ability to operate the robot remotely to respond to emergencies or perform actions that the robot had not yet learned to be both useful and necessary. However, they preferred the robot to be automated for most tasks. One participant stated the following:

It’s good to have the opportunity to do that [distant operating], but then, you should be told that “now we can see into your home.”

Participant 4

Another said the following:

Yes, so it has to have that function...well there will always be some challenging tasks, there are no homes or institutions that are the same, so there may be physical things that make it possible for it to mess up.

Participant 2

When discussing how the robot could be useful for fulfilling their needs, the participants that received assistance in personal care stated that the robot could never replace their human carers completely. By watching the robot in action, they realized that it could not assist them in tasks such as using a breathing mask or catheterization. One participant stated that, the previous year, she could have benefitted from the practical assistance that was demonstrated by the robot, but as her function had deteriorated, especially her breathing and the risk of having something stuck in her throat, she could only be without her human helpers for very short periods of the day.

Several of the other participants stated that they already had assistants or home services attending to most of their needs. Still, the participants sometimes experienced pressure ulcers or infections, which forced them to bed rest. They suggested having the robot present during these periods to assist in fulfilling the tasks they needed.

However, most participants agreed that such a robot could replace some of their assistants’ time and be particularly useful in the gaps when they did not have a human helper:

As a backup, they [the assistants] are out driving a lot with the kids to training and so on, and they are shopping for me, it’s nice for me to be at home, but during summer I have to drink a lot of water, so then it could be nice [to have a robot assisting].

Participant 5

#### Ease of Use

Through the interviews, it appeared that the participants had above-average technological knowledge. They had advanced wheelchair controls, smart house functions, and different technical aids. None of the participants expressed any worries about being able to control the robot. All agreed that voice control was preferred. Some stated that a combination of voice and gestures and lights would be fine. All agreed that the robot would need to express some kind of confirmation on having received and understood a command, preferably through sound as the robot could be out of its user’s sight at the time:

But you never know if the robot has confirmed or not...you need to know that it has received...if it repeats what it is supposed to do...that it confirms that it has received, for example when I ask for a glass of water, it should say “yes, it has been received, I will fetch a glass of water.”

Participant 2

One participant also commented that the robot’s eyes and face should follow its users for more normalized communication. The participants expressed that the speakers and microphone for EVE would need to be of a better quality. All wanted a humanlike voice for the robot and the ability to choose the voice of the robot. One participant expressed that, similarly to his smart home control, he expected the robot to learn his voice and that he would be able to extend commands to the robot and they would learn from each other.

#### Perceived Adaptiveness

The participants expressed that the robot would need to be adapted to their particular needs. As most of the participants had reduced ability to grip objects, they were concerned with the robot handing over objects and particularly waiting to release the grip until they had managed to get a grip around the item. They mentioned the need for the robot to adapt to be able to navigate around the users’ homes. Some were concerned with the robot’s ability to understand the different Norwegian dialects:

So if...you can go in...and teach it commands, that you can go in and type in commands and then it will execute it, I think in Norway where are so many dialects, you have to find a way that you can override that [the programming]...so that the robot can teach itself.

Participant 2

Another participant stated the following:

*[About receiving an object from the robot]It worked out really well. I was very unsure about when it would release the grip, because I was unsure if the operators were watching...I would like to have it like...if I said “ok,” then it would let go, that it was sort of an agreement.* [Participant 3]

### Personal Norms

#### Privacy and Trust

Seven participants stated that they were not concerned at all with any privacy issues regarding having the robot in their homes. Even though they had been informed about how the robots used cameras for navigation and that remote operators would be able to connect through the robot, they did not find this disturbing. However, they did appreciate the idea of a sound or light announcing that someone was connecting from the operating central. Two participants were concerned about the idea that someone “looked” into their house without being informed. They suggested regulations that illegalized this and a log that could be checked for any distant use. One participant stated the following:

I haven’t thought about privacy at all, because I would never communicate any details to such a machine anyway that I couldn’t handle being exposed.

Participant 1

Another said the following:

It’s kind of important that it [the robot] manages to do things automatically in relation to privacy...and so that no one sits looking into your house all the time, yes, because then you’re alone, then you know that, this is my place and there’s no one else lurking around.

Participant 4

All of the participants stated that they would trust the robot. One mentioned that he would trust it if he had a log to check for distant on-logging. They related this both to privacy issues and also physical safety. One participant said the following:

It will be the same as an alarm company...I have an alarm here...they can connect to the cameras at any time in theory, but you have to have confidence in it...and if someone abuses this, it must be prosecuted, so it’s about trust and that you have legislation if something illegal was going to happen.

Participant 2

#### Autonomy and Independence

The participants had varying views on whether such a robot would increase their independence or autonomy. Most (n=7) believed that the robot could enhance their sense of freedom by assisting with necessary tasks, allowing them to do what they wanted, whenever they wanted, without waiting for assistants, home nurses, or family members. Two participants felt that the absence of a human caregiver and reduced need for constant human interaction would be advantages of robotic assistance. In addition, 2 participants mentioned that having robotic support during times without human assistance would make them feel safer rather than providing a feeling of autonomy:

Yes exactly, I think it’s very fascinating and it’s also a bit...because I get a little tired in my head from having people here all the time because...I cannot help but feel like being at “work” in a way, that I feel I have to be in such a nice positive mode and do some nice things [for and with the assistants]. Because even though I know that it is not in the job role of personal assistants, it is my life that should be lived the way I want to live it, but I have to practice that extremely much, to completely relax when there are other people in the house. Yeah, I wish I could zero out my head and I think I would have done that easier with a robot than a human in a way.

Participant 9

### Social Norms

#### Social Influence

Within this subcategory participants shared how others’ opinions on whether they had a robot as an assistant mattered to them. Four participants stated that others’ opinions would not affect their decision to have robotic assistants as they did not care about others’ opinions. Two participants were concerned about others’ opinions, especially on the robot’s physical appearance in the room when having visitors:

...it matters a little to me if they [guests] feel alright in room.

Participant 9

Three participants stated that, somehow, they were often affected by others’ opinions, but this was not particularly connected to robotic assistance. They cared about whether others had opinions on having assistance in general and on using technical aids:

Yes, it matters to me what people think, whether it’s robots or assistants or the wheelchair, so of course you have that in the back of your mind, but from experience, so...knowledge and exposure tend to solve a lot of it.

Participant 2

Another participant stated something similar:

I’ve actually thought the same about having assistants...yes that “he’s in such a poor condition that he must have assistants,” they’d probably think that it was very strange [to have robotic assistance].

Participant 7

However, most participants believed that opinions about robotic assistance would not be negative, whereas one participant was concerned that health care employees might worry about losing their jobs.

#### Societal Impact

In addition to discussing the robot as a potential assistant for themselves, the participants also talked about how the robot could be useful for others. Several of the participants were concerned about the challenges in health care and how this might affect access to help for people in need of assistance and saw robots as a natural part of future health care services. One stated that the home nurses were so busy all the time and it would be good if robots could take some pressure off them, acknowledging that robotic assistance might not involve direct care but could instead focus on meeting practical assistance needs:

I am very positive to the idea that health professionals should be relieved of all the running...not necessarily the care...like wound care, but ask if you’re okay and so on...and serving breakfast or taking a breakfast order.

Participant 3

#### Sociability

The participants discussed the robot’s communication abilities and whether these abilities were important to them. For instance, several of the participants stated that they felt that it was natural to be polite to the robot and thank it for its services. They attributed this to its human likeness. As mentioned previously, one participant expressed dissatisfaction with the robot’s inability to make eye contact during communication, suggesting that eye contact would make the interactions feel more natural. Most participants indicated that they did not prefer or need to engage in extensive communication with the robot beyond issuing commands as they preferred to fulfill their social needs with other humans. However, some noted that social interaction with the robot could be beneficial for individuals who live alone or feel isolated.

### Control Beliefs

#### Anxiety

None of the participants reported feeling general anxiety or fear during the interactions. At one point, the robot moved behind the participants to lower a curtain, which they could hear but not see. Despite this, none of the participants expressed feeling uneasy. However, some participants described the robot’s appearance and movements as “somewhat frightening,” “a little creepy,” or “not particularly nice looking.” One stated the following:

Yes, but it was really only when it came in there and did that stuff, when it took that blanket and bent down it was almost like a snake person...and it doesn’t say anything...just completely silent, just some ventilators running that’s what was a bit creepy.

Participant 7

Another participant said the following:

No, it wasn’t uncomfortable at all, but I wondered what he was going to do, but then I looked to the side, and I saw the blind coming down, then he pulled it all the way down.

Participant 6

#### Safety

The participants reported feeling safe in the robot’s presence, and it was suggested that the robot should be able to alert health services in case of an emergency. The participants who had children in the house did not feel particularly concerned about the robot causing any harm; they simply stated that they expected the robot to be safe before implementation. One participant stated the following:

No, not at all [felt unsafe] and it was because I saw that it was so steady on the wheels, I analyzed it before it started driving. Another thing is that if something were to happen that there could have been a connection so that he [the robot] could have notified someone, to call someone at the home-services station: “now you have to come.”

Participant 1

### Facilitating Conditions

Regarding physical facilitating conditions, all participants stated that their home was already wheelchair accessible, so both placing the robot and the robot moving around was not seen as a problem:

.*..if people have a wheelchair, then it is very adapted for people who are on wheels, so in that sense, it is almost better that the robot is on wheels than if it should walk.* [Participant 2]

This was supported by another participant:

Yes, it’s good to have houses and apartments for wheelchair users because there are few thresholds, there are open solutions and things are kind of in such a way that it’s easily accessible...I have my technical room around the corner there [I would have placed it there], but then it’s a bit far away, so then it would take 20 seconds before he came into place, but if there are some automatic tasks, then it’s ok.

Participant 3

Three participants expressed that they would prefer to have the robot placed in a separate room—to keep it out of sight—just as they did with other technical aids. Some participants were curious about the organization of a robot service. They expressed concerns that, if each robot required a separate remote operator, it would affect the possible societal impact.

### Intention to Use

Although the participants were impressed by the robot’s functions and its ability to perform tasks aligned to their needs, they did not see the robot as ready for implementation. However, they were willing to test the robot in their own home and were positive to using it when the technical readiness had progressed to a point at which the robot could perform most assistance tasks they needed autonomously. All participants saw the robot as a service to be combined with other assistance, and they would accept someone operating the robot from a distance if it was necessary. The mistakes that the robot made during the interactions, such as dropping an object or the fan being very loud at times, did not affect their willingness to have the robot at home. Some participants said the following:

...as long as it correct its mistakes.

Participants 3, 4, and 6

Another stated the following:

I can do that, I would be willing to try [the robot as a home service]...but of course I wouldn’t have it instead of the care that the home care service comes with, I wouldn’t replace that, no. So, care in the bathroom and care with inserts and things like that, I wouldn’t have wanted to use a robot for those kind of tasks.

Participant 6

## Discussion

### Principal Findings

The aim of this study was to investigate the usefulness, ease of use, and acceptance of the prototype humanoid assistive robot EVE among users with physical disabilities, framed around key concepts from the Almere model and the model of DSRA: hedonic attitudes, utilitarian attitudes, personal norms, social norms, control beliefs, and facilitating conditions [27,28].

Our methodology used directed content analysis, initially applying a deductive approach to analyzing statements from participant interactions with the robot EVE. Subsequently, we adopted an inductive strategy, anticipating the discovery of statements misaligned with concepts from the Almere and DSRA models given their focus on assistive and domestic social robots for users without disabilities. Contrary to our expectations, the frameworks by Heerink et al [27] and de Graaf et al [28] aligned quite well with participant feedback, underscoring the relevance of these models even in contexts involving assistive robots for individuals with physical disabilities. However, we modified the subcategory Attractiveness to encompass the subcategories Appearance and Animacy. Participants were less concerned with the robot’s attractiveness but more with its appearance, discussing issues such as size, speed, design, and anthropomorphism. Opinions on the robot’s size varied among participants. Some felt that the robot was too large and appeared masculine, whereas others appreciated its ability to reach objects at higher levels. Most of the participants emphasized its humanlike appearance without it being unsettling. They preferred functional aspects over a fully humanlike appearance to avoid a “creepy” effect. This sense of discomfort has been termed the “uncanny valley” by Mori et al [55]. They noted that robots with humanlike features are generally more appealing to people but only to a certain extent. Beyond the uncanny valley, people’s attraction turns into unease and a propensity to feel frightened.

While participants primarily valued the robot’s assistive capabilities over its potential for entertainment, several noted that interacting with the robot was enjoyable and anticipated that having it at home would be fun. Hedonic factors such as appearance and enjoyment are crucial for acceptance and have been shown to directly influence the intention to use a robot [27,56]. Although robots designed for physical assistance are primarily utilitarian, they also incorporate hedonic aspects. Even if these hedonic elements are only partial, enjoyment remains a critical construct in any acceptance model for robotic technology.

The functionality of the robot impressed nearly all participants, especially its ability to perform precise tasks such as picking up and handling objects without spillage. Participants valued the robot’s potential to assist with personalized tasks, although they recognized that it could not replace their human caregivers for more sensitive tasks. Participants also expressed ideas about how others might use the robot and noted that its deployment could potentially alleviate the burden on health care staff, highlighting its societal impact. Most participants in this interaction study had previously participated in a focus group that explored user needs and requirements after viewing videos of the robot EVE [15]. Although their identified tasks that would make the robot useful were similar to those mentioned in the focus group study and fell within the “Activity and Participation” category of the ICF framework [15,53,54], we observed that their expectations of the robot significantly increased after seeing it in real life. Studies on this issue present mixed results, revealing that users’ expectations can either differ or remain consistent across virtual and real-life encounters with robots [57-59]. Real interaction may demystify a technology, making it more approachable and less intimidating. Once participants witnessed firsthand what the robot could do, their comfort with the technology may have grown, consequently raising their expectations. Direct interaction exposes users to the robot’s practical applications and capabilities, potentially surpassing their initial assumptions based on mere descriptions or videos. This exposure could shift their expectations as they begin to envision more ways in which the robot could be beneficial in their daily lives. Moreover, after experiencing the robot’s assistance, participants might recognize that it can perform tasks more effectively or efficiently than anticipated. This realization leads them to expect more from future interactions, seeing the robot’s potential more clearly and imagining broader or more sophisticated applications for its use. In addition, the fact that the tasks performed by the robot were personalized specifically to meet their needs and executed exactly as the participants had described may have enhanced the overall experience.

Participants familiar with various technologies expressed confidence in their ability to operate (ease of use) the robot, favoring voice control as the primary mode of interaction. Although the robot made some mistakes, such as freezing for some seconds or dropping an object, the participants did not react negatively to this, stating that this would be expected of novel technology. This finding is different from those of other studies whose participants became frustrated with the robots’ errors [60,61]. It is likely that this could be related to age or previous experiences with technology. This highlights the importance of designing robots with effective error management and recovery strategies to minimize frustration and enhance user interaction.

The consensus on the necessity for robots to adapt to specific user needs highlights a critical area of development in assistive robotics [22]. This adaptation is particularly crucial in tasks such as handling objects with care and effectively navigating home environments. Traditionally, the capacity for robots to adapt to the unique needs of individual users has been seen as overly complex and cost prohibitive. The challenges stemmed from the need for sophisticated sensing technologies and algorithms capable of understanding and responding to a diverse range of human behaviors and environmental contexts. However, recent advances in AI have begun to shift this perspective. AI technologies, particularly those involving machine learning and deep learning, have made significant strides in enabling robots to learn from interactions and adapt their behaviors over time. It will now be possible to analyze vast amounts of data generated from user interactions to continually refine and improve robot performance according to specific user preferences and needs. The integration of AI allows for greater personalization in robotic systems, which has been highlighted as crucial for adoption in several studies [22]. This can include adjusting the grip strength when handling objects or enhancing the robot’s ability to operate in complex environments, such as typical home settings where every space is unique.

To our surprise, privacy concerns were minimal among most participants despite their awareness of the robot’s data collection capabilities. They expressed a prominent level of trust in the robot, equating its use to that of other everyday technologies such as smartphones and home alarm systems. Participants also indicated that they were accustomed to the frequent presence of visitors in their homes, which may have contributed to their relaxed attitude toward privacy. During discussions about remote operation, participants preferred the robot to handle tasks autonomously. However, they acknowledged the necessity for remote intervention in situations in which the robot had not yet learned a specific task or during emergencies. de Graaf et al [28] discovered that lower levels of privacy concerns were associated with a higher likelihood of using a robot.

Although robots may be a means to solve some of the future health care challenges, we believe that the primary goal of using a physically assistive robot is to support the user’s sense of autonomy and independence. Therefore, we included questions on this topic in our interviews despite its absence as an influential factor in existing literature on the acceptance of social robots. Previous studies have emphasized the importance of being able to act independently, doing what one wants, when one wants [15]. Participants have also highlighted the significance of not feeling like a burden to caregivers and the value of experiencing freedom from human carers occasionally [15]. We suggest including concepts on independence and autonomy when investigating the acceptance of robots assisting with daily activities.

Participants expressed varied opinions on the significance of others’ perceptions regarding their use of robots as home assistants. Some were indifferent, whereas others cared to a degree similar to the degree to which they cared about others’ reactions to other forms of assistance or technical aids. When interacting with the robot, participants were polite, stating that this behavior came naturally; however, they emphasized that their primary expectation of the robot was for physical assistance, not for engaging in conversation. Social norms play a crucial role in shaping individuals’ attitudes toward robots by setting expectations for their behavior and societal acceptance [28]. These norms both directly and indirectly affect one’s willingness to adopt and integrate robots into daily life. In addition, the degree to which robot use is viewed as normal or endorsed by one’s social circle significantly influences an individual’s decision to use robots [28].

Although participants noted that the robot appeared large and masculine, they felt safe around it and experienced no anxiety during their interactions. They also expressed no concern about the facilitating conditions as their living facilities were already adapted for wheelchair use.

### Methodological Strengths and Limitations

The acceptance models developed by Heerink et al [27] and de Graaf et al [28] have predominantly been applied to quantitatively assess the acceptance of robots, particularly among older populations. In our study, we adapted concepts from these models to guide a qualitative investigation. We contend that using qualitative methods is particularly advantageous when seeking deeper, more nuanced insights into the reception of novel technologies, such as the humanoid robot EVE used in this research. This approach allows us to capture the rich, detailed user experiences and perceptions that quantitative methods might overlook, providing a comprehensive understanding of how individuals interact with and react to advanced robotic technologies in real-world settings.

The use of purposive sampling in this study might have introduced bias by including participants particularly interested in robotic assistance, which could have influenced their generally positive attitudes toward the technology [15]. We recognize that the small sample size of 9 participants may be seen as a limitation. However, we believe that the information power was sufficient, as defined by Malterud et al [62], who suggest that such power is achieved when participants actively share their experiences, thereby fulfilling the study’s objectives. Our participants, all of whom had physical disabilities and varied levels of dependency on assistance, were engaged and provided rich, insightful discussions about the potential of the robot as a novel health care service. This depth of qualitative data bolstered the study’s information power. In addition, our findings were rigorously analyzed through a 16-step process described by Assarroudi et al [41], resulting in categories that align with existing theories on robotic acceptance, directly addressing the research questions and aims of the study [27,28,41].

A significant number of participants had experienced an SCI, which could have introduced a specific bias in our results. Despite this, the needs expressed were strikingly consistent across different disabilities, focusing on functionality over the nature of the disability, such as impaired upper-limb function. Moreover, the sample included more male participants, mirroring the demographic trends observed in stroke and SCIs. Despite this gender imbalance, there were no notable differences in the needs or acceptance between male and female participants [63,64].

Using the Wizard of Oz approach raises an ethical concern due to the element of deception as participants are often unaware that a human operator is controlling the robot. This lack of transparency can raise issues related to trust and honesty in human-robot interactions. However, a study by Nasir et al [65] explored the impact of revealing the “wizard” behind the robot and found that participants’ perceptions of the robot’s autonomy remained largely unchanged even after the deception was disclosed. While the Wizard of Oz method remains a valuable tool for simulating and testing robot behavior, managing deception carefully is crucial, particularly in long-term human-robot interaction studies.

### Conclusions

The results of our study reveal a complex interplay of functional expectations, technological readiness, and personal and societal norms influencing the acceptance and intended use of a physically assistive robot among individuals with physical disabilities. Participants were generally positive about the potential of robotic assistance to enhance their independence and reduce reliance on human caregivers for certain tasks, albeit recognizing the robot’s current limitations and future potential. While the robot was not deemed ready for immediate full-scale implementation, participants were open to further testing in their homes, with the condition that the robot could perform most tasks autonomously in the future.

For developers, this study underscores the importance of a human-centered, iterative design process. The feedback collected has already informed improvements, leading to the development of a smaller, more functional robot with humanlike fingers and enhanced automation, better tailored to individual user needs. Health care managers, on the other hand, should recognize that, while robotic assistance is progressing, its integration into care settings will require significant adjustments to existing services. This includes rethinking care delivery models, staff training, and the balance between human and robotic caregivers.

As we move closer to successfully integrating robotic assistance into users’ homes, both developers and health care managers should prepare for future implementation. This preparation includes addressing system maintenance, remote operation, and service delivery. The next steps should involve larger-scale studies to evaluate these aspects in real-world care environments.

Overall, the application of AI in robotics opens up new possibilities for creating more adaptable and personalized assistive technologies. This progress could reduce the costs associated with personalized adaptations but also enhance the effectiveness of assistive robots, making them a more viable and helpful option for individuals with disabilities.

## Appendix

Multimedia Appendix 1 Demonstration of 5 tasks for participant 9—shortened.

Multimedia Appendix 2 Semistructured interview.

Multimedia Appendix 3 Concepts with definitions.

## Abbreviations

AI: artificial intelligence
DSRA: domestic social robot acceptance
HCD: human-centered design
ICF: International Classification of Functioning, Disability, and Health
PAI: physical artificial intelligence
SCI: spinal cord injury
