# Supplementary material for: User Acceptance of a Home Robotic Assistant for Individuals With Physical Disabilities: Explorative Qualitative Study
Source: JMIR Rehabil Assist Technol. 2025 Jan 13;12:e63641. doi: 10.2196/63641 (PMC11758889; doi:10.2196/63641)
Supplement: Multimedia Appendix 2 [file rehab_v12i1e63641_app2.pdf]

Semistructured interview

The interview was conducted in Norwegian. (Translation by Google translate, with personal adjustments for publication in an international journal).

Research questions:	***1. What is the perceived usefulness, ease of use and user acceptance of the humanoid robot EVEr3 to help in daily life activities, among users with physical disabilities?***<br><br>***2. How do users think a humanoid robot will affect their independence and sense of autonomy?***
Generally	• **You have now experienced the robot in action. Can you tell us about your experiences with this trial?**<br>• **What do you think you could have wanted help with from this robot?**
Anxiety	• **What do you think it would be like to have such a robot at home? (negative/positive)**
Facilitating conditions/ Intention to Use	• **What conditions would have to be present if you were to have the robot in your home? (if you would need help from others to use it, if you would have to make adjustments at home)**
Perceived Ease of Use	• **What do you think it will take for you to be able to use it?**<br>• **Do you think it would be easy or difficult for you to control the robot? How?**
Perceived adaptiveness	• **What would it take for you to use the robot at home over time?**
Perceived Enjoyment/attractiveness/anxiety	• **How would you describe the robot in your own words?**
Perceived Sociability	• **What do you think it will be like to interact and communicate with the robot? What would be important to you in terms of communicating with it?**
Perceived Usefulness	• **Do you think the robot might be useful to you?**<br>• **How can the robot be useful for you?**
Social Influence	• **What do you think those around you think about you having and using such a robot?**<br>• **What does it mean to you what others think?**
Trust	• **What would it take for you to trust such a robot?**
Privacy and safety	• **What do you think about privacy and security when using such a robot? (description of the terms)**

Attitude	• **What do you think about the fact that the health service will use such a robot to assist users?**
Independence, coping and Autonomy	• **How do you think that the help of such a robot can affect your independence in everyday life?**<br>• **How do you think that the help of such a robot will affect your freedom to live life the way you want?**
