# Supplementary material for: User Acceptance of a Home Robotic Assistant for Individuals With Physical Disabilities: Explorative Qualitative Study
Source: JMIR Rehabil Assist Technol. 2025 Jan 13;12:e63641. doi: 10.2196/63641 (PMC11758889; doi:10.2196/63641)
Supplement: Multimedia Appendix 3 [file rehab_v12i1e63641_app3.pdf]

Concepts with definitions used in analysis

Concepts	Descriptions/definitions of the concepts from the literature, own understandings/definitions in *italic*.	Questions often used from Almere (1) or DSRA (2) models to investigate the concepts
ANX Anxiety	Evoking anxious or emotional reactions when it comes to using the system.<br>*Or not*<br>*May overlap with attractiveness, trust and safety*	**Almere**<br>"If I should use the robot, I would be afraid to make mistakes with it. If I should use the robot, I would be afraid to break something. I find the robot scary. I find the robot intimidating"
**ATT Attitude towards technology.**	Positive or negative feelings about the appliance of the technology<br>*In general, i.e. personal or for the healthcare sector- included in **Societal Impact***	**Almere**<br>"I think it's a good idea to use the robot. The robot would make life more interesting. It's good to make use of the robot"
FC Facilitating conditions	Factors in the environment that facilitate use of the system.<br>*Physical in the house/apartment, next of kin*	**Almere**<br>I have everything I need to use the robot. I know enough of the robot to make good use of it
ITU Intention to Use	The intention to use the system over a longer period in time	**Almere, DSRA**<br>"Assuming I have a robot, I will frequently use it in the future"
PAD Perceived adaptiveness	The perceived ability of the system to adapt to the needs of the user<br>*i.e personalization*	**Almere, DSRA**<br>I think a social robot would be adaptive to what I need"
PENJ Perceived Enjoyment	Feelings of joy/pleasure associated with the use of the system	**Almere, DSRA**<br>"I would enjoy a social robot talking to me
PEOU Perceived Ease of Use	The degree to which one believes that using the system would be free of effort<br>*If they will manage to use it, communication, interface*	**Almere, DSRA**<br>"I think I would know quickly how to use a social robot"
PS Perceived Sociability	The perceived ability of the system to perform sociable behavior<br>*Will it function well in understanding, communication, including with visitors*	**Almere, DSRA**<br>"A social robot would feel comfortable in social situations"

**PU Perceived Usefulness**	The degree to which a person believes that the system would be assistive<br>*Including in what it will or will not be useful for*	**Almere, DSRA**<br>“I think a social robot would be useful to me”
**SI Social Influence**	The persons perception that people who are important to him think he should or should not use the system	**Almere, DSRA**<br>“People would find it interesting to use a social robot”
**SP Social Presence**	The experience of sensing a social entity when interacting with the system	**Almere, DSRA**<br>“Interacting with a social robot would feel like interacting with an intelligent being”
**Trust**	The belief that the system performs with personal integrity and reliability<br>*If the user trusts it to be reliable, may overlap with safety and privacy*	**Almere, DSRA**<br>“A social robot should be: dishonest … honest”
**Cost**	The extent to which the current cost of a (PC) robot is too high (Venkatesh and Brown 2001 (3)).<br>*Or comments on price, personal and for society*	**DSRA**<br>I think social robots would be quite pricy
**Safety**	Perceived safety describes the user’s perception of the level of danger when interacting with a robot, and the user’s level of comfort during the interaction (Bartneck et al 2009(4))	**DSRA**<br>Being near a social robot would make me feel: anxious … relaxed
**Self-efficacy**	An individual's belief in his or her capacity to execute behaviors necessary to produce specific performance attainments (Bandura 1977 (5)).<br>*Overlaps with ease of use?*	**DSRA**<br>“I would be able to use a social robot<br>if someone showed me how to do it first”
**Societal Impact**	*This term is not defined in the referenced literature, but is an overarching term that deals with the influence of robots on people, social interactions and society (including economy)*<br>*Including participants general attitudes of the robot as an assistive technology*	**DSRA**<br>“I feel that society will be dominated by robots in the future”
**Privacy**	Information privacy refers to the claim of individuals, groups or institutions to determine for themselves when and how and to what extent information about them is communicated to others (Westin 1967 p 7 in Malhotra et al 2004 (6))	**DSRA**<br>“It would bother me if I had to give personal information to a social robot”
**Status**	Whether users believe a social robot; when they perceive that having such a robot enhances of affects their social status (de Graaf Moore 2017 (2), Moore and Benbasat 1991 (7))	**DSRA**<br>“People who would own a social robot would have more prestige than those who do not”

**Attractiveness**	Interpersonal attraction, a multidimensional construct, a social or personal liking property; a physical dimension based on dress and physical features; and a task-orientation dimension related to how easy or worthwhile working with someone would be (McCroskey & McCain 1974 (8))	**DSRA** “I think a social robot would look quite pretty”
**Companionship**	*No definition in the referenced literature, but deals with the extent to which users want "companionship" with the robot (McCroskey & McCain 1974 (8)) (i.e: companionship with social robots is a complex interplay of emotional, social, cognitive, and ethical elements, aiming to create beneficial interactions between humans and robots Kidd, C. D., & Breazeal, C. (2008), (9)Breazeal, C. (2003)) (10)*	**DSRA** “I would be able to establish a personal relationship with a social robot
**Animacy**	Animacy is, according to the Oxford Dictionary defined as “having life, lifely” (Bartneck et al 2009 (4)). *Whether the robot should look, sound or behave human-like or not. Can also mean animal-like but in this study, we focus on human-like*	**DSRA** “A social robot would be: dead … alive”
**Independence**	Concerning functioning independently in ADL, functioning without assistance (Wade and Colin 2009). (11) *We include this term to investigate whether participants believe the robot could contribute to independence*	**BARTHEL**
**Autonomy**	The principle of autonomy (literally, self-rule) freedom to make their own choices and to act on the basis of such choices (Kersten et al 2006 (12)). *We include this term to investigate whether participants believe the robot will contribute to autonomy*	**IPA**

DSRA: A Model of Domestic Social Robot Acceptance. IPA: Impact on participation and autonomy questionnaire. Concepts in green were used in the study.

1. Heerink M, Krose B, Evers V, Wielinga B. Assessing Acceptance of Assistive Social Agent Technology by Older Adults: the Almere Model. I J Social Robotics. 2010;2:361-75.
2. de Graaf MMA, Ben Allouch S, van Dijk JAGM. Why Would I Use This in My Home? A Model of Domestic Social Robot Acceptance. Human–Computer Interaction. 2019;34(2):115-73.

3. Venkatesh V, Brown SA. A Longitudinal Investigation of Personal Computers in Homes: Adoption Determinants and Emerging Challenges. MIS Quarterly. 2001;25(1):71-102.
4. Bartneck C, Kulić D, Croft E, Zoghbi S. Measurement Instruments for the Anthropomorphism, Animacy, Likeability, Perceived Intelligence, and Perceived Safety of Robots. International Journal of Social Robotics. 2009;1(1):71-81.
5. Bandura A. Self-efficacy: Toward a unifying theory of behavioral change. Psychol Rev. 1977;84(2):191-215.
6. Malhotra NK, Kim SS, Agarwal J. Internet Users' Information Privacy Concerns (IUIPC): The Construct, the Scale, and a Causal Model. Information Systems Research. 2004;15(4):336-55.
7. Moore GC, Benbasat I. Development of an Instrument to Measure the Perceptions of Adopting an Information Technology Innovation. Information Systems Research. 1991;2(3):192-222.
8. McCroskey JC, McCain TA. The measurement of interpersonal attraction. Speech Monographs. 1974;41(3):261-6.
9. Kidd CD, Breazeal C, editors. Robots at home: Understanding long-term human-robot interaction. 2008 IEEE/RSJ International Conference on Intelligent Robots and Systems; 2008 22-26 Sept. 2008.
10. Breazeal C, Dautenhahn K, Kanda T. Social robotics. Springer Handbook of Robotics2016. p. 1935-71.
11. Collin C, Wade DT, Davies S, Horne V. The Barthel ADL Index: a reliability study. Int Disabil Stud. 1988;10(2):61-3.
12. Kersten P, Cardol M, George S, Ward C, Sibley A, White B. Validity of the impact on participation and autonomy questionnaire: a comparison between two countries. Disabil Rehabil. 2007;29(19):1502-9.
